# Spatial Transcriptomics and Dual Dye Mapping Identify Wnt-Driven BBB Protection in Endothelial EphA4-Deficiency

**DOI:** 10.21203/rs.3.rs-9261231/v1

**Published:** 2026-04-09

**Authors:** Caroline de Jager, Jing Ju, Marco Corbo, Karan Patel, Michelle Theus

**Affiliations:** Virginia Tech

**Keywords:** traumatic brain injury (TBI), Eph signaling, neuroprotection, spatial transcriptomics, brain microvasculature, blood-brain barrier (BBB)

## Abstract

Blood-brain barrier (BBB) disruption is a key pathological event following traumatic brain injury (TBI), yet its molecular and spatial characteristics remain incompletely understood. Here, we developed a dual dye-labeling system to assess the temporal and spatial dynamics of BBB permeability following controlled cortical impact (CCI) injury in *EphA4f/f VE-Cadherin-CreERT2* (KO) and *EphA4f/f* (WT) mice. By tracking Evans Blue Dye (EBD), sodium fluorescein (NaFl), and IgG deposition, we reveal distinct patterns of extravasation in the injured cortex and hippocampus. NaFl, a small-molecule tracer, continues to extravasate for 7 days post-injury, whereas EBD leakage diminishes after 4 days. Notably, EC-specific EphA4 KO mice exhibit a protective role in BBB integrity. To further investigate BBB regulation, we integrated spatial transcriptomics with dye quantification, revealing that EphA4 EC ablation upregulates key BBB-related genes (*Tjp2, Tjp3, Cldn1, and Ocln*) and neuroprotective genes (*Nr4a1* and *Npas4*). Notably, Wnt signaling genes are upregulated in the KO cortex, and we demonstrate that inhibition of Frizzled-4 (FZD4)/Wnt attenuates BBB protection in KO mice. Importantly, direct pharmacological activation of Wnt signaling with the FZD4 agonist FZM1.8 reduces lesion volume and BBB disruption. Overall, these findings demonstrate the effectiveness of spatial transcriptomics and dual-dye labeling in uncovering region-specific transcriptional changes associated with BBB disruption following CCI injury and in assessing the influence of EphA4/Wnt signaling. Wnt signaling emerges as a promising pathway for BBB protection and repair following TBI, offering a potential strategy to mitigate secondary brain injury.

## Introduction

Traumatic brain injury (TBI) is a leading cause of neurological dysfunction, with effects that extend well beyond the initial insult. While mild TBI (commonly referred to as a concussion) often resolves quickly, moderate to severe TBIs can lead to persistent neurological deficits and an increased risk of long-term complications (Haarbauer-Krupa et al., 2021). Beyond the immediate consequences, secondary injury cascades, characterized by oxidative stress, neuroinflammation, and blood-brain barrier (BBB) disruption, exacerbate acute recovery and set the stage for long-term functional decline. (Khatri et al., 2021). The BBB plays a critical role in maintaining brain homeostasis and is a primary target of injury-induced pathophysiological changes. Disruption of the BBB following controlled cortical impact (CCI) injury leads to increased vascular permeability, immune cell infiltration, and neuroinflammatory signaling, all of which contribute to secondary tissue damage. Despite its importance, the molecular mechanisms underlying BBB dysfunction and repair following TBI remain poorly characterized, particularly in a spatially defined context.

Remarkable progress in describing cell states, both healthy and diseased, has been made using RNA sequencing approaches (Stark et al., 2019). For example, single-cell RNA sequencing (scRNAseq) was first described in 2009 (Tang et al., 2009) and has enabled the exploration of cellular heterogeneity following TBI (Arneson et al., 2018; Garza et al., 2023; Qiu et al., 2023). However, a key limitation of scRNA-seq is the loss of spatial resolution, which makes it difficult to capture the spatial organization of heterogeneous cell populations and molecular hallmarks in the injured brain. Addressing this gap, spatial transcriptomics provides a critical approach for mapping gene expression in situ, enabling a more comprehensive understanding of region-specific BBB dysfunction and cellular responses following TBI. Understanding BBB disruption at high spatial resolution is essential for characterizing region-specific effects of TBI in preclinical animal models, enabling more rigorous design and assessment of BBB-targeted therapeutics (Obenaus & Badaut, 2022; Yen et al., 2023). Localized assessments of BBB integrity may improve the mechanistic understanding of changes in vascular permeability that may drive regional disruptions of the injured milieu, including inflammation, neuronal function, and metabolic dysregulation, ultimately contributing to long-term neurological impairment (Ya et al., 2023).

Historically, BBB disruption has been studied using a number of tools, including Evans’ Blue Dye (EBD) absorption readings, quantitative PCR, and western blotting to probe expression changes, IgG deposition, and blood biomarkers (such as Occludin). However, these methods have significant limitations. EBD primarily binds to serum albumin, giving it a large molecular weight (~ 67 kDa)(Yao et al., 2018), which restricts its ability to detect subtle changes in BBB permeability. Additionally, chemical extraction methods used for EBD quantification can introduce variability by binding to proteins, thereby skewing absorbance readings (Grimbleby & Ntailianas, 1961). Despite these challenges, there remains a critical need for improved methodologies that enable quantitative, region-specific assessments of BBB extravasation. Traditional techniques often lack spatial precision, making it difficult to map BBB permeability across heterogeneous brain regions following injury. To overcome these limitations, we developed a dual-dye labeling system using i.v. injection of sodium fluorescein (NaFl; <1kDa) and EBD (69kDa) alongside IgG staining (150kDa), combined with spatial transcriptomics to quantify regional extravasation and gene expression changes at high resolution (Duan et al., 2023).

Spatial sequencing provides a comprehensive view of the molecular landscape of the injured brain across different anatomical regions that display altered dye extravasation (Zheng et al., 2023). Importantly, we test this using endothelial cell (EC)-specific EphA4 KO mice, which our prior research has implicated as a key mediator of tissue damage and BBB disruption(Cash et al., 2023; Frugier et al., 2012). Eph receptors and their ephrin ligands are bidirectional signaling partners(Xu & Henkemeyer, 2012) that have been linked to the CNS response to injury and neurodegenerative conditions (Malik & Di Benedetto, 2018). By integrating spatial transcriptomics with functional dye-based BBB permeability assays, this study provides a high-resolution molecular atlas of BBB disruption and repair following TBI, highlighting EphA4 as a critical modulator of injury-induced vascular dysfunction and Wnt signaling as a promising therapeutic target for mitigating BBB damage and secondary brain injury.

## Results

### Spatial and Temporal Quantification of BBB Disruption in the CCI-Injured Murine Brain.

To assess the acute and subacute dynamics of BBB permeability, wild-type (WT) mice underwent controlled cortical impact (CCI) injury, and dye extravasation into the injured cortex was quantified (Supplemental Fig. 1). One hour prior to perfusion, mice were tail vein injected with Evans Blue Dye (EBD, 67 kDa) and sodium fluorescein (NaFl, 376 Da), allowing circulation throughout the vasculature. Cardiac perfusion was then performed to clear serum, endogenous proteins, and unbound dye, ensuring that extravasated dye represented BBB permeability (Supplemental Fig. 1A).

Following perfusion, brains were serially cryosectioned at 30 μm spaced 450 μm apart and processed for IgG immunostaining (IgG, ~ 150 kDa) to further assess vascular permeability to endogenous proteins. Confocal images were acquired (Supplemental Fig. 1B–H), and quantification of IgG, EBD, and NaFl extravasation was performed using the Cavalieri probe in StereoInvestigator (MBF Bioscience). Representative images from 1-, 4-, and 7-days post-injury (dpi) show distinct temporal patterns of dye and protein extravasation (Supplemental Fig. 1F–H). At 1 dpi, extravasation of IgG, EBD, and NaFl is most pronounced, indicating significant BBB permeability (Supplemental Fig. 1F). By 4 dpi, EBD and IgG extravasation are markedly reduced, whereas NaFl extravasation remains similar to 1 dpi, suggesting sustained permeability to smaller molecules (Supplemental Fig. 1G). By 7 dpi, EBD is fully excluded from the brain parenchyma, NaFl extravasation persists, indicating a selective recovery of the BBB to large molecules while remaining permeable to smaller solutes (Supplemental Fig. 1H). The conclusion of the study at 7 dpi was chosen as previous research indicated the resolution of BBB permeability to EBD at this stage(Cash et al., 2023). By 14 dpi, NaFl was excluded from the injured cortex (Supplemental Fig. 1I-1K). These findings reveal a time-dependent, size-selective BBB repair process, in which large proteins such as IgG and EBD are cleared more rapidly, while the permeability of small molecules remains disrupted beyond 7 dpi.

### Endothelial Cell (EC)–Specific EphA4 Ablation Reduces Overall Expression and Alters Temporal BBB Dynamics After CCI-Induced Brain Injury.

**Figure 1:**
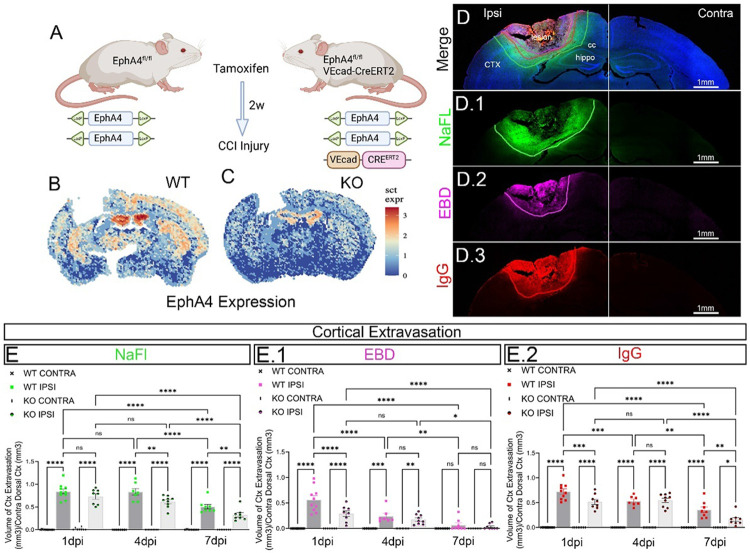
Endothelial Cell–Specific EphA4 Ablation Reduces Overall Expression and Alters Temporal BBB Dynamics After CCI-Induced Brain Injury. (A) Schematic representation of the genetic strategy used to generate endothelial cell-specific EphA4 knockout (KO) mice. EphA4^fl/fl^ mice were crossed with VE-cadherin (VEcad)-CreERT2 mice to enable tamoxifen-inducible deletion of EphA4 in endothelial cells. Two weeks after tamoxifen administration, mice underwent CCI injury for subsequent analysis. (B–C) Spatial transcriptomic analysis of EphA4 expression in wild-type (WT). (B) and endothelial-specific EphA4 knockout (KO). (C) brain sections following CCI injury. Heatmaps indicate relative EphA4 transcript levels, with warmer colors representing higher expression. Loss of endothelial EphA4 results in a marked reduction in overall EphA4 expression, particularly in the hippocampus, cortex, and thalamus. (D-D.3) Representative confocal images of cortical extravasation at 4 dpi, showing merged signals (D), NaFl (D.1, green), EBD (D.2, magenta), and IgG (D.3, red). (D–E.2) Quantification of cortical extravasation of NaFl, (E); EBD, (E.1); and IgG (E.2) at 1-, 4-, and 7-days post-injury (dpi) in wild-type (WT) and EC-specific EphA4 knockout (KO) mice. Ipsilateral (IPSI) and contralateral (CONTRA) hemispheres are compared. Scale bar: 1 mm. n=8-10. Statistical significance was determined using two-way ANOVA; Bonferroni post-hoc. Data presented as mean ± SEM; **p < 0.01, ***p < 0.001, ****p < 0.0001, ns = not significant.

EC-derived EphA4 has been identified as a mediator of TBI sequelae. To investigate its role in post-injury vascular dysfunction, we subjected *EphA4*^*f/f*^*/VE-Cadherin-Cre*^*ERT2*^ (KO) and *EphA4*^*f/f*^ (WT) mice to CCI injury. Adult male EphA*4* ^*f/f*^*/VE-Cadherin-Cre*^*ERT2*^ and *EphA4* ^*f/f*^ mice at 8 weeks of age were intraperitoneally injected with tamoxifen (50 mg/kg) for 5 consecutive days to induce Cre-mediated recombination in ECs. After a 2-week washout period, which allowed for recombination and clearance of tamoxifen metabolites, mice underwent CCI injury (Fig. 1A). To confirm successful recombination and reduction of EphA4 expression in KO tissues, we employed spatial transcriptomics (SpatialGE) (Ospina et al., 2025; Ospina et al., 2024; Ospina et al., 2022) to visualize EphA4 transcript distribution across brain sections. WT mice exhibited robust EphA4 expression, particularly in vascular-rich regions (Fig. 1B), whereas KO mice showed a marked reduction in EphA4 transcripts, confirming effective endothelial-specific deletion (Fig. 1C). These findings establish a model for investigating the role of EC-specific EphA4 in BBB integrity following TBI and lay the groundwork for further analyses on its downstream effects on vascular permeability and regional responses.

To assess the acute and subacute dynamics of BBB permeability, we quantified EBD, NaFl, and IgG extravasation in the contralateral (CONTRA) and ipsilateral (IPSI) cortices of WT and KO mice at 1-, 4-, and 7-days post-injury (dpi) (Fig. 1D–1E). NaFl extravasation was consistently greater than EBD at all time points, reflecting size-dependent differences in BBB permeability (Supplemental Fig. 2). While EBD extravasation was significantly elevated in the injured cortex at 1 dpi and 4 dpi, it was no longer significant at 7 dpi, indicating progressive BBB recovery (Fig. 1E.1). In contrast, NaFl continued to extravasate at 7 dpi, suggesting sustained permeability to smaller molecules (Fig. 1E). IgG deposition followed a similar trend, showing a significant decrease between 1 dpi and 4 dpi, but no further reduction at 7 dpi, indicating that while large proteins were gradually cleared, BBB permeability remained partially compromised (Fig. 1E.2). As expected, little to no extravasation was observed in the contralateral cortex across all markers and time points (Fig. 1D–1E.2).

We next quantified dye and protein extravasation in EphA4 KO and WT mice to evaluate the role of endothelial EphA4 in BBB disruption (Fig. 1E–1E.2.2). NaFl extravasation was significantly reduced in KO cortices at 4 dpi and 7 dpi compared to WT, indicating enhanced BBB integrity in KO mice (Fig. 1E). However, no differences were observed at 1 dpi, suggesting that early BBB permeability was comparable between genotypes. EBD and IgG extravasation were significantly lower in KO cortices at 1 dpi, but differences in IgG were only significant at 7 dpi, suggesting that the loss of endothelial EphA4 accelerates recovery of BBB integrity (Fig. 1E.1–E.2).

Although the primary lesion was cortical, dye and protein extravasation extended to the hippocampus, where regional differences between WT and KO mice were also evident (Supplemental Fig. 3A–3C.2). This is of particular interest as hippocampal function is disrupted post-TBI (Baskaya et al., 1997; Beamer et al., 2016; Rola et al., 2006; Zhang et al., 2020). EBD extravasation increased in WT tissue between 4 and 7 dpi but showed no differences between WT and KO (Supplemental Fig. 3C.1). IgG deposition decreased in WT hippocampi between 1 dpi and 4 dpi, also showing no differences between WT and KO (Supplemental Fig. 3C.2). NaFl extravasation was significantly reduced in KO tissue compared to WT at 1 dpi and 4 dpi but showed no difference at 7 dpi (Supplemental Fig. 3C). Representative images of hippocampal extravasation at 1 dpi in WT and KO mice highlight these differences (Supplemental Fig. 3A.1–3A.4, Supplemental Fig. 3B.1–3B.4). These findings demonstrate that EC EphA4 deletion reduces BBB permeability over time, particularly for smaller molecules such as NaFl, which may accelerate recovery following CCI injury.

### Spatial Transcriptomics Reveals EphA4-Dependent Gene Expression Changes and Pathway Enrichment in the Injured Cortex.

**Figure 2:**
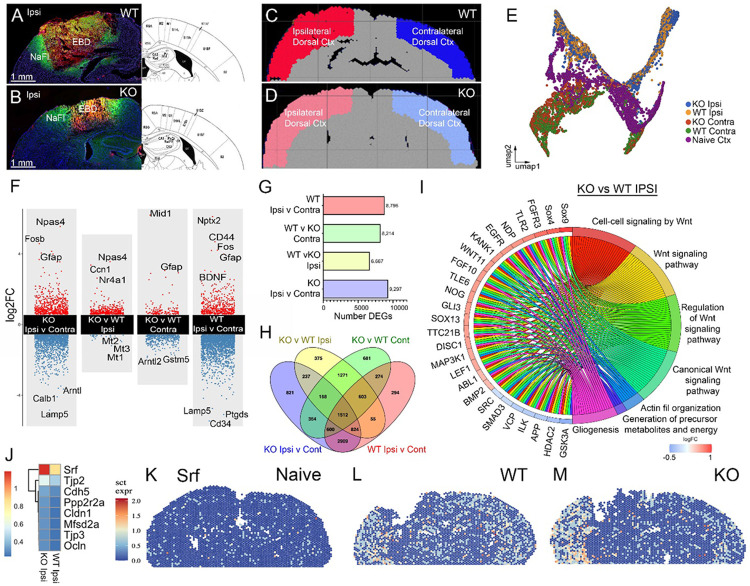
Comparative spatial sequencing analysis of the damaged cortex in WT and KO mice 1 dpi. (A, B) Ipsilateral coronal sections of the brain from WT and KO mice, respectively, showing a representative confocal image of EBD (magenta), NaFl (green), and DAPI (blue). The images illustrate differences in dye extravasation between WT and KO mice. (C, D) Annotated clusters of the ipsilateral and contralateral somatosensory cortex (SSC) in the WT and KO dorsal cortex, respectively. (E) UMAP showing clustering of spatial sequencing data from different brain regions in WT and KO mice. Each color represents a different region: KO Ipsilateral cortex (blue), KO Contralateral cortex (red), WT Ipsilateral cortex (yellow), WT Contralateral cortex (green). (F) Transformed volcano plot showing differentially expressed genes between different cortical regions, highlighting significant genes such as *Gfap*, *Bdnf*, *Mid1*, and *Npas4*. (G) Bar graph displaying the number of differentially expressed genes across cortical regions and conditions. (H) Venn diagram illustrating the overlap of differentially expressed genes among four cortical comparisons between WT and KO. (I) Circular chord plot depicting signaling pathways enriched in differentially expressed genes in KO versus WT Ipsilateral cortex, revealing the Wnt signaling pathway enriched in KO cortex. (J) Heatmap of genes important for BBB function and integrity. (K-M) Feature of Srf in the brain tissue of Naïve WT (K), WT CCI (L), and KO CCI (M) mice.

To assess transcriptional differences between injured and uninjured cortical tissue, we utilized Visium spatial transcriptomics (10x Genomics). Regions of interest (ROIs) in the dorsal cortex at 1 dpi of WT and KO CCI-injured mice were analyzed using the Partek^©^ classification workflow (excluding the motor cortex) (Fig. 2A–2D). Uniform Manifold Approximation and Projection (UMAP) visualization demonstrated clear clustering of naïve, injured, and uninjured cortical regions, supporting distinct transcriptional profiles based on injury status (Fig. 2E). Differential gene expression (DGE) analysis in WT mice revealed upregulation of well-known injury-associated genes, including *Fos, Gfap, CD44*, and *Bdnf* in the injured cortex compared to uninjured tissue, reflecting glial activation and neuroinflammatory responses (Fig. 2F) (Dragunow & Robertson, 1988; Matzilevich et al., 2002; Pekny & Pekna, 2004).

To investigate the role of EphA4 deletion in injury response, we compared gene expression between the KO and WT injured cortex (Fig. 2F–H). In KO tissue, *Npas4* and *Nr4a1*, genes associated with neuroprotection and synaptic plasticity, were significantly upregulated following injury (Fig. 2F) (Choy et al., 2016; Wu et al., 2019). Conversely, *Mt1, Mt2*, and *Mt3*, key regulators of metal homeostasis and oxidative stress responses, were downregulated in KO tissue (Fig. 2F). The total number of differentially expressed genes (DEGs) across comparisons ranged from 6,667 to 9,297 (Fig. 2G), with 1,512 DEGs overlapping across all four comparisons, highlighting conserved molecular injury responses and EphA4-dependent effects (Fig. 2H). Gene ontology (GO) enrichment analysis in KO vs. WT injured cortex identified pathways related to Wnt signaling, actin filament organization, gliogenesis, and metabolic precursor generation, suggesting that EphA4 deletion modulates injury-induced molecular pathways with potential implications for BBB repair and neuroprotection (Fig. 2I). The heatmap highlights a number of genes involved in BBB integrity and function which are upregulated in the KO injured tissue (Fig. 2J). Serum response factor (*Srf*), a transcription factor involved in BBB integrity, was upregulated in the KO dorsal injured cortex compared to the WT injured cortex (Fig. 2L and 2M), and both WT and KO injured cortices showed greater expression than the WT Naïve tissue (Fig. 2K) (Weinl et al., 2015).

### Spatial Transcriptomics Reveals Region-Specific Gene Expression Changes Following BBB Disruption and EC-specific EphA4 Deletion.

**Figure 3:**
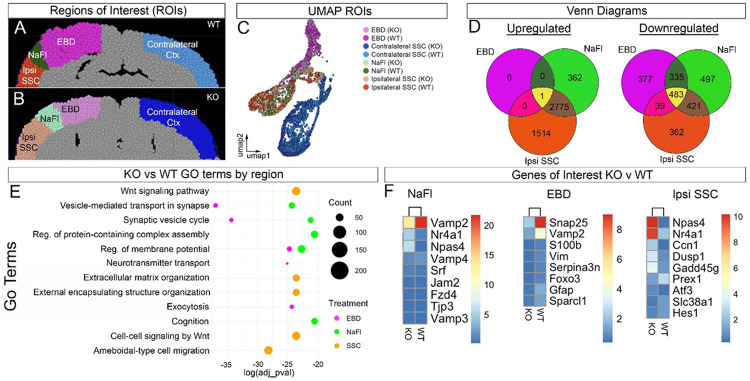
Spatial and molecular heterogeneity of transcriptomic changes across dye-labeled regions in EC-specific EphA4 KO mice. (A, B) Regions of Interest (ROIs) for EBD and NaFl in the brain are shown of KO and WT CCI-injured mice. The regions include EBD and NaFl-stained ipsilateral cortex, contralateral somatosensory cortex (Contralateral SSC), and ipsilateral somatosensory cortex (Ipsi SSC). (C) UMAP displaying the clustering of different ROIs based on gene expression profiles obtained through spatial sequencing. Each color represents a different ROI for each genotype: Naïve, EBD, NaFl, contralateral SSC, and Ipsi SSC. (D) Venn diagrams illustrating the number of upregulated and downregulated genes in each ROI when comparing KO to WT mice. (E) Gene Ontology (GO) enrichment analysis comparing KO to WT across EBD, NaFl, and SSC brain regions. The dot plot indicates significant GO terms related to biological processes, including vesicle-mediated transport in the synapse, regulation of the postsynaptic membrane potential, and others. Dot size represents the number of genes from the input list that are annotated to a given GO, while color indicates the brain region. (F) Heatmaps showing specific DEGs of interest in KO and WT mice across three ROIs: NaFl, EBD, and Ipsi SSC. Notable genes include Vamp2, Npas4, Nr4a1, Foxo3, and Hes1, among others, which regulate transcytosis.

After establishing a spatial transcriptomic model to analyze injured brain tissue, we compared transcriptional differences between distinct dye- and IgG-labeled regions relative to the uninjured contralateral cortex (Fig. 3A–3B). EBD and NaFl extravasation served as a guide for defining regions of interest (ROIs). Uniform Manifold Approximation and Projection (UMAP) visualization revealed distinct clustering of gene expression profiles across the EBD, NaFl, ipsilateral (no dye), and contralateral (uninjured) cortical regions, supporting unique transcriptional identities associated with vascular permeability and injury response (Fig. 3C).

Venn diagram analysis showed the number of overlapping differentially expressed genes (DEGs) between KO and WT tissues across these regions, highlighting region-specific transcriptomic shifts (Fig. 3D).

To further characterize biological pathways altered in the absence of endothelial EphA4, we performed Gene Ontology (GO) analysis of DEGs in the EBD and NaFl regions. GO enrichment revealed distinct molecular processes disrupted in each dye region, including post-synapse organization, regulation of membrane potential, and extracellular matrix organization, indicating that dye-based ROI selection corresponds to unique transcriptional changes following injury (Fig. 3E).

Lastly, several key genes were differentially expressed between KO and WT tissues in each ROI (Fig. 3F). In the NaFl region, genes associated with transendothelial migration (*Vamp2, Vamp4*) were downregulated, while *Nr4a1*, a neuroprotective transcription factor, was upregulated. In the EBD region, *Vim* (vimentin), *Gfap* (glial fibrillary acidic protein), and *Snap25* (synaptic vesicle protein) were downregulated in KO tissue, suggesting a potential reduction in astrocytic reactivity and synaptic remodeling. In the ipsilateral somatosensory cortex (SSC), Nr4a1 was upregulated, further reinforcing its potential role in EphA4-mediated neuroprotection. These findings indicate that distinct BBB permeability regions correspond to region-specific transcriptional changes, and that EphA4 deletion may influence synaptic organization, neuroprotection, and astrocyte reactivity following CCI injury.

### EC-specific EphA4 Deletion Alters Regional Dye-Specific Gene Expression and Highlights Synaptic Pathways Following CCI Injury.

**Figure 4:**
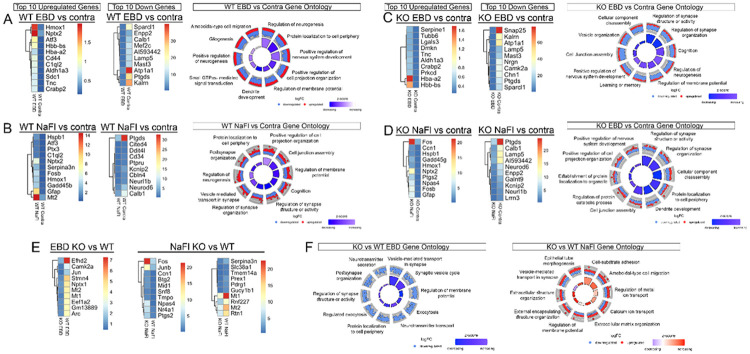
Top genes and comparative gene ontology (GO) analysis across dye regions in EC-KO compared to WT injured mice. (A) GO enrichment analysis comparing EBD vs control, highlighting significant biological processes and pathways affected. (B) GO enrichment analysis comparing NaFl vs control, showing the impact on various cellular functions. (C) KO EBD vs control GO ontology, illustrating the differential gene expression and associated biological processes. (D) KO NaFl vs control GO ontology, depicting changes in gene expression and relevant biological pathways. (E) KO vs WT EBD and NaFl comparison, indicating significant alterations in gene function between knockout and wild-type samples. (F) KO vs WT EBD and NaFl GO ontology, summarizing the differences in gene expression and functional categories. Overall, the highest-enriched KO bp categories include synaptic signaling, vesicle-mediated transport, inflammatory responses, and blood-brain barrier regulation.

To characterize transcriptional differences between ipsilateral and contralateral regions, we performed spatial transcriptomics in WT and KO mice and analyzed DEGs in EBD- and NaFl-defined regions (Fig. 4A–4D). In WT EBD regions, we observed upregulation of hemoglobin-related genes (*Hbb-bs, Hba-a2*) and downregulation of synaptic transmission genes (*Sparcl1*, *Ptgs2*), suggesting a shift toward injury-induced metabolic and structural reorganization (Fig. 4A). GO analysis revealed a reduction in neurogenesis and neuron signal transduction pathways, further supporting injury-induced impairments (Fig. 4A). Similarly, WT NaFl regions showed upregulation of pro-inflammatory and immediate early response genes (*Gfap, Ptx3, Atf3, Fosb*) and downregulation of memory-associated genes (*Calb1, Cd34*), with GO analysis indicating potential disruptions in synaptic organization and neurogenesis (Fig. 4B) (Goffigan-Holmes et al., 2019).

Likewise, the KO EBD regions showed similar gene expression trends with increased hemoglobin-related genes (*Hba-a2, Hbb-bs*) and decreased synaptic transmission genes (*Snap25, Camk2a, Sparcl1*) (Fig. 4C). GO analysis showed a potential reduction synaptic function and neurogenesis, indicating that these pathways may remain vulnerable in the core damage region even in the absence of EC-specific EphA4 (Fig. 4C). In KO NaFl regions, we observed upregulation of genes associated with astrocyte activation (*Gfap*), immediate early gene expression (*Fos, Npas4, Gadd45g*), alongside downregulation of memory-associated genes (*Calb1, Lamp5*) (Fig. 4D). GO analysis suggested alterations in genes involved in synaptic activity and structure (Fig. 4D).

Interestingly, when comparing KO vs. WT EBD regions, we identified downregulation of genes involved in cytoskeletal dynamics, vesicular transport, calcium signaling, and metabolic function (*Efhd2, Camk2a, Stmn4, Nptx1*), suggesting potential disruptions in neuronal signaling and BBB stability (Fig. 4E). GO analysis highlighted pathways potentially involved in synaptic structure, synaptic vesicles, neurotransmitter secretion and membrane potential (Fig. 4F), suggesting decreased signaling involved in excitotoxicity. On the other hand, NaFl regions in KO vs. WT showed enrichment of genes, *Fos, Junb, Npas4*, and *Nr4a1*, associated with microglial activation, BBB integrity, and neuroprotection, while several downregulated genes are involved in neuroinflammation and GABAergic transmission pathways (*Serpina3n, Slc38a1*) (Fig. 4E). Gene ontology analysis suggested KO mice show regionals differences including an increase in pathways involved in extracellular matrix organization and regulation of membrane potential, and a decrease in vesicle- mediated transport at the synapse (Fig. 4F). By leveraging spatial transcriptomics, we identified divergent molecular responses in EBD- and NaFl-defined extravasation zones, highlighting distinct injury-induced processes.

### Spatial transcriptomics in EC-specific EphA4 KO mice identifies Wnt signaling as a therapeutic target for BBB repair following CCI injury.

**Figure 5:**
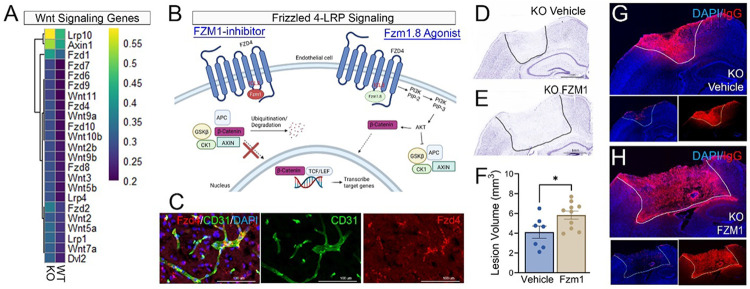
Modulation of Wnt signaling in EphA4 KO endothelium influences BBB integrity and lesion volume following CCI injury. (A) Heatmap of Wnt signaling gene expression highlighting differential expression of key components in WT and EC-specific EphA4 KO ipsilateral cortex. Color intensity corresponds to relative expression levels. (B) Schematic representation of Fizzled-4 (FZD4)–LRP5/6 signaling pathway, and associated downstream effectors (e.g., GSK3β, β-catenin). (C) Representative max-z projection confocal image of immunofluorescence showing Fzd4 (red) and CD31 (green) co-localization in brain vasculature, indicating endothelial expression of Wnt-related signaling components. (D, E) Nissl-stained brain tissue from KO mice treated with vehicle or FZD4 inhibitor, FZM1. Quantification in (F) reveals a significant increase in lesion volume in FZM1-treated KO mice (n=7-10, t-test; *p* < 0.05). (G-H) IgG extravasation in KO mice treated with vehicle (G) or FZM1 (H). Dashed lines delineate regions of IgG leakage, indicative of BBB disruption.

Spatial RNA sequencing revealed increased expression of Wnt signaling genes in KO ipsilateral cortical tissue compared to WT, suggesting that EphA4 deletion enhances Wnt pathway activation (Fig. 5A). Given Wnt signaling’s established role in BBB and blood-retinal barrier (BRB) maintenance (Laksitorini et al., 2019; Wang et al., 2020; Zhang et al., 2023), we investigated its functional relevance in BBB integrity following CCI injury. Canonical Wnt signaling is initiated by the binding of low-density lipoprotein receptor-related protein (Lrp) and Frizzled (Fzd) receptors to Wnt ligands, leading to the recruitment of Axin, CK1, and GSK3β to phosphorylated Lrp. This cascade stabilizes β-catenin, allowing it to translocate to the nucleus and activate transcription of BBB-regulatory genes, such as Cldn5, which are essential for barrier function (Fig. 5B). Fzd4 expression primarily colocalizes with CD31 + endothelial cells (Fig. 5C).

To determine whether EC EphA4 deletion-mediated Wnt activation is protective, we first inhibited Wnt signaling in KO mice using the Fzd4 inhibitor Fzm1 at 1 dpi. BBB disruption was greatest at 1 dpi; therefore, Wnt signaling was targeted at this stage. Fzm1 acts as a negative allosteric modulator of Fzd4 by binding to an allosteric binding site located in intracellular loop 3, ultimately altering the conformation of the Fzd4 receptor (Riccio et al., 2018). KO mice treated with Fzm1 exhibited larger lesion volumes and greater IgG deposition, indicating attenuation of BBB protection compared to vehicle-treated KO mice (Fig. 5D–5H).

**Figure 6:**
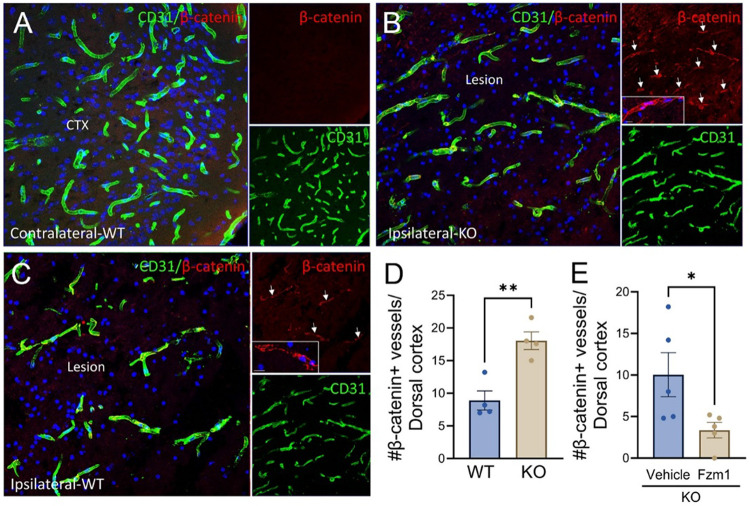
Loss of endothelial cell (EC) EphA4 increases vascular β-catenin expression, which is attenuated using Frizzled 4 (FZD4) receptor inhibitor Fzm1 after 1 day CCI injury. (A-C) Representative confocal images of CD31+ (green) and β-catenin+ (red) vessels in the WT contralateral (A), KO ipsilateral (B), and WT ipsilateral (C) cortices at 1 dpi. (D) Quantification of β-catenin+/CD31+ vessels in the WT and KO ipsilateral cortex. (E) Quantification of β-catenin+/CD31+ vessels in the KO ipsilateral cortex after vehicle or Fzm1 treatment at 1 dpi. Statistical significance was determined using an unpaired t-test. Data presented as mean ± SEM; T-test in D and E. *p < 0.05, **p < 0.01.

β-catenin expression on blood vessels in the injured cortex was increased in the ipsilateral KO (Fig. 6B, 6D) compared to WT mice (Fig. 6C, 6D). Furthermore, β-catenin expression on blood vessels in the Fzm1-treated KO injured cortex was decreased compared to vehicle-treated KO mice (Fig. 6E).

**Figure 7:**
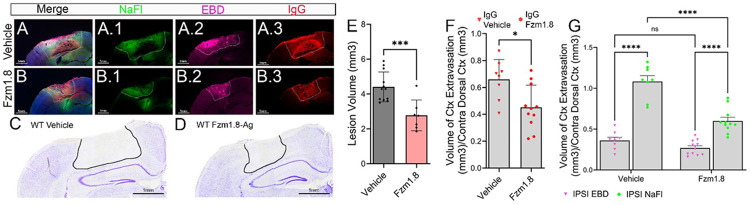
Wnt signaling activation using Frizzled 4 (FZD4) receptor agonist protects BBB integrity and reduces lesion volume. (A-A.3; B-B.3) Effects of FZD4 agonist (Fzm1.8) on BBB permeability. Merged images and separate channels show NaFl (green), EBD (magenta), and IgG (red), showing BBB impairment under vehicle conditions and protection with Wnt activation. (F-G) Quantification of extravasation volume in vehicle control versus Fzm1.8-treated groups, showing significantly reduced NaFl and IgG leakage with Wnt agonists (n=8-12, two-way ANOVA; ****p < 0.0001, *p < 0.05, ns = not significant). (C-E) Lesion volume quantification showing a significant decrease with Fzm1.8 treatment compared to control (n=7-11; unpaired t-test, ***p < 0.001). (C-D) Representative Nissl images, corresponding to quantitative measures in (E).

Next, we examined whether directly enhancing Wnt signaling could promote BBB repair using a Wnt agonist, Fzm1.8 (Fig. 7A–7G). Fzm1.8 is an allosteric agonist of FZD4 via activation of the Wnt/β-catenin pathway via PI3K signaling, thereby promoting TCF/LEF transcriptional activity (Riccio et al., 2018). Since FZD4 expression is primarily restricted to endothelial cells (Fig. 5C), these agonists allowed us to selectively enhance Wnt signaling in the cerebrovasculature. Analysis of dual-dye labeling showed that Fzm1.8 treatment significantly reduced NaFl and IgG extravasation compared to vehicle controls (Fig. 7A–7B and 7F–7G, respectively), confirming enhanced BBB integrity following Wnt activation after TBI. Moreover, lesion volume was significantly reduced at 1 dpi in Fzm1.8-treated mice (Fig. 7C–7E), further supporting the role of Wnt signaling in mitigating secondary injury and promoting BBB repair. These findings suggest that EphA4 negatively regulates Wnt signaling after CCI injury, and that pharmacological activation of Wnt enhances BBB integrity and reduces tissue damage, highlighting Wnt signaling as a potential therapeutic target for post-TBI BBB repair.

## Discussion

These novel findings provide new insights into the use of spatial sequencing to reveal region-specific transcriptional changes associated with BBB disruption across molecular-weight-restricted, dye-labeled regions in the presence or absence of EC-specific EphA4 following CCI injury. EBD-labeled regions, representing areas of severe BBB disruption with high-molecular-weight extravasation, exhibited potential downregulation of synaptic transmission pathways and reduced neurogenesis, suggesting widespread neuronal dysfunction and synaptic destabilization. Conversely, NaFl-labeled regions, which remained permeable to smaller molecules at later time points, were enriched in genes associated with neuroinflammation, extracellular matrix remodeling, and membrane potential regulation, reflecting a sustained but more moderate disruption in BBB integrity. This distinction may prove important for the development of small versus larger molecule drugs in the future, and the time frame during which they are administered. When comparing WT and KO tissue, endothelial EphA4 deletion altered these region-specific transcriptional landscapes, showing synaptic and neurogenesis-related impairments persisted, but astrocyte reactivity markers (e.g., *Gfap, Vim*) were reduced compared to WT, suggesting a muted glial response. In KO NaFl regions, BBB integrity appeared more preserved, as evidenced by upregulation of neuroprotective genes *(Nr4a1, Npas4*) and downregulation of neuroinflammatory markers (*Serpina3n, Slc38a1*) compared to WT.

These findings demonstrate that spatial sequencing enables a nuanced understanding of BBB dysfunction by identifying distinct molecular consequences in regions with differential permeability, and that EphA4 deletion modulates neuroinflammation and BBB recovery in a region-specific manner. It is particularly valuable for understanding the complex, heterogeneous nature of TBI, which can affect various brain regions in different ways (Piwecka et al., 2023), as TBI does not uniformly affect the entire brain (McAllister, 2011). The injury’s impact can vary across regions, leading to distinct molecular, cellular, and tissue-level changes (Arneson et al., 2022; Jha et al., 2024). Spatial sequencing enables researchers to map gene expression and protein markers within specific brain regions and structures, providing a highly detailed view of how different areas are affected by injury. This level of precision helps identify injury hotspots and regions that may show signs of neurodegeneration or inflammation, which could inform future therapeutic strategies.

The dual dye-labeling method also revealed that EBD and NaFl extravasation into the ipsilateral cortex varied over time, with NaFl persisting through 7 days post-injury, while EBD extravasation was no longer significant by day 7, indicating size-dependent differences in BBB permeability. IgG deposition remained present for up to a week, decreasing between 1- and 7-days post-injury. One limitation of this study is that dye extravasation quantifications were concluded at 7dpi. Future studies may investigate NaFl extravasation beyond 7dpi to determine when the BBB is no longer compromised to small molecules. EphA4 KO mice exhibited reduced NaFl extravasation at 4 and 7 dpi, suggesting a protective role for EphA4 deletion in mitigating prolonged BBB disruption, with NaFl effectively distinguishing BBB recovery at later time points. Our findings also reveal the effects of CCI injury on hippocampal BBB disruption over the acute time course. It remains poorly understood whether disruptions to vascular integrity led to long-term neurocognitive deficits, as serum proteins such as fibrinogen can chronically accumulate in the brain, potentially contributing to persistent neuroinflammation and cognitive decline (Arciniegas et al., 2002; Iggena et al., 2023; Milner, 1972; Patel et al., 2024; Rempel-Clower et al., 1996; Scoville & Milner, 2000; Skelton et al., 2000; Sulimai & Lominadze, 2020). Importantly, it is well recognized that TBI-induced BBB disruption is associated with worse clinical outcomes and cognitive decline (Che et al., 2024; Yang et al., 2021).

Maintaining barrier integrity and regulating tight junctions are crucial for the proper function of the BBB. We find that genetic ablation of endothelial EphA4 increases the expression of key BBB genes, including *Tjp2*, *Tjp3*, *Cdh5*, *Ocln*, and *Cldn1*. *Mfsd2a*, a key mediator of BBB function, was also increased in the KO. *Ppp2r2a*, a gene known to be involved in tight junction trafficking, was also upregulated. Moreover, we find that EC-specific EphA4 KO cortex shows key upregulated genes, including Npas4 and Nr4a1, both of which have been shown to have neuroprotective roles in brain injury (Choy et al., 2016; Rothe et al., 2017; Wu et al., 2019). Notable downregulated genes included *Mt1*, *Mt2*, and *Mt3*. *Mt1* is a known regulator of the Wnt signaling pathway, facilitating reduced β-catenin translocation into the nucleus and potentially preventing the transcription of BBB genes (Dai et al., 2021).

Wnt signaling is a crucial regulatory pathway involved in various aspects of cell function, including development, tissue repair, and cellular homeostasis. It plays a significant role in the development, maintenance, and function of the BBB (Hussain et al., 2022; Laksitorini et al., 2019; Liebner et al., 2008). In the context of BBB integrity, Wnt signaling helps regulate the formation and maintenance of tight junctions between endothelial cells (Laksitorini et al., 2019). The canonical Wnt pathway, in which Wnt ligands bind to cell-surface receptors (Lrp and Fzd), activates intracellular signaling cascades that lead to the stabilization of β-catenin (Liu et al., 2022). This, in turn, promotes the expression of genes involved in endothelial cell function and tight junction integrity, such as *Cldn5*, a critical component of tight junctions. This study explored Wnt signaling as a potential therapeutic target for disrupting the BBB. Wnt pathway activation in WT mice using an agonist (Fzm1.8) led to a reduction in lesion volume and less BBB disruption, suggesting that modulating Wnt signaling may be a promising approach to mitigating BBB damage following brain injury. A limitation of this study was the administration of Wnt-modulating drugs only at 1 dpi, as the goal was to establish a basic link between EphA4 and Wnt signaling. Future studies will aim to elucidate the mechanism by which EphA4 modulates downstream Wnt signaling, as well as Wnt signaling modulation at later time points. Other agonists have been described for protecting the BRB [51] and the BBB in a stroke model (Ding et al., 2023).

In summary, this study demonstrates the utility of spatial transcriptomics combined with dual-dye labeling in identifying region-specific transcriptional responses to BBB disruption following CCI injury. Severely disrupted EBD regions exhibited synaptic dysfunction and neuroinflammatory activation, whereas NaFl regions showed prolonged but more selective BBB compromise; EphA4 deletion enhanced barrier integrity by upregulating tight junction and neuroprotective genes while reducing glial reactivity. Furthermore, our findings highlight the therapeutic potential of activating Wnt signaling for BBB repair, as pharmacological modulation of Wnt signaling reduced lesion volume and mitigated BBB disruption. Future studies should explore the translational potential of Wnt agonists for TBI therapy and further elucidate the interplay between EphA4 and Wnt signaling in endothelial cells.

## Methods

### Animals.

All mice were housed and bred in an AAALAC-accredited facility with a 12-hour light-dark cycle, and food and water were provided ad libitum. For the experiments, 7 to 12 male wildtype floxed *EphA4*^*f/f*^ or *EphA4*^*f/f*^/*VE-Cadherin-Cre*^*ERT2*^ mice on the CD1 background, aged 8 to 10 weeks, were used. All procedures followed the NIH Guide for the Care and Use of Laboratory Animals and were approved by the Virginia Tech Institutional Animal Care and Use Committee (IACUC; #24–041) and the Virginia-Maryland College of Veterinary Medicine. Sex as a biological variable: Male mice were used to minimize variability from hormonal fluctuations associated with the female estrous cycle, which could affect vascular and immune responses. This choice improves reproducibility and aligns with prior studies. Future work will include both sexes to assess sex-specific effects. EphA4 knockout experiments used *EphA4*^*fl/fl*^ (WT) and *EphA4*^*fl/fl*^*/VECadherin-Cre*^*ERT2*^ (KO) were bred on CD1 background for a minimum of 10 backcrosses. Adult male *EphA4*^*f/f*^ and *EphA4*^*f/f*^*/VE-Cadherin-Cre*^*ERT2*^ mice at 8 weeks of age received intraperitoneal injections of tamoxifen (Sigma Aldrich; St. Louis, MO) at a dose of 50mg/kg for five consecutive days. Two weeks after tamoxifen administration, genotyping was performed to verify Cre expression and EphA4 flox excision, and CCI injury was induced. The following primers were used for genotyping: FA4FLOX2: TAATTGTAATCAGTGGGCGGGC; F10718flox: GCA CAC TTA GCA ATT CAG TGT GGG; R10719flox: CCT GCA AAT TAA GGG CAG GAA GAG; oIMR1084(Cre3): GCG GTC TGG CAG TAA AAA CTA TC; oIMR1085 (Cre4): GTG AAA CAG CAT TGC TGT CAC TT.

### Controlled cortical impact (CCI) injury.

Male mice were anesthetized with a pre-mixed ketamine (100 mg/kg) and xylazine (10 mg/kg) cocktail via subcutaneous injection and positioned in a stereotaxic frame, and CCI was performed as previously described(Kowalski et al., 2019) using Φ = 3-mm round tip connected to an eCCI-6.3 device (Custom Design & Fabrication, LLC) at a velocity of 5.0 m/s, depth of 1.8mm ± 0.05mm, and 150ms impact duration. Sham animals underwent craniotomy only. For Fig. 7, immediately after CCI, mice received a bolus injection of 10 μg/g of Fzm1.8, 10 μg/g of Fzm1, or vehicle via i.v. tail vein injection (MedChemExpress, Monmouth Junction, NJ, Fzm1.8: HY-117163, Fzm1: HY-116553). Each animal was coded, and the experimenter was blinded from group and experimental conditions. A total of 88 mice were used for this study, with the overall mortality rate of the procedure being 3.40% (3 of 91 mice). Therefore, 88 were included in the data presented within this publication. Inclusion criteria consisted of ensuring body weights falling between 30 and 50 grams. Exclusion criteria included reopening of incisions, unusual behavior from the animal, poor i.v. tail vein injections of drugs or dyes, and insufficient or severe CCI injury due to user error. Outliers were determined using FDR.

### Blood-Brain barrier permeability analysis.

We measured BBB as previously described (Brickler et al., 2018). Briefly, a 2% sterile Evans Blue Dye (EB, Sigma E2129) and 20% Sodium Fluorescein (NaFl, F1272) solution was i.v. tail vein injected (4 mL/kg of body weight), and one-hour post-injection, the brains were perfused with 4% PFA, then post-fixed for 4hrs in PFA. Serial coronal sections were rinsed with 1X PBS, then blocked in 2% cold water fish skin gelatin (Sigma, Inc.) and 0.2% Triton-X100 for 30 minutes. Slides were incubated with a secondary antibody for IgG staining and mounted in media containing DAPI counterstain (SouthernBiotech, Birmingham, AL). The secondaries used were all raised in donkey (Thermo Fisher Scientific, USA). Mounted tissue sections were imaged on a Nikon A1 confocal microscope using a 4x objective. Volume of extravasation (mm^3^) in the ipsilateral and contralateral cortex was assessed using the Cavalieri method with StereoInvestigator software (MicroBrightField, Williston, VT, USA) on an upright Olympus BX51TRF motorized microscope (Olympus America, Center Valley, PA, USA). The analysis was performed blindly using the confocal images of five serial coronal sections spaced 15 slices apart (−1.1 to −2.9 mm A/P). The data are presented as the volume of tissue damage normalized by the contralateral cortex or hippocampus (mm^3^).

### Lesion Volume Analysis.

Lesion volume (mm^3^) in the ipsilateral cortex was assessed using the Cavalieri method with StereoInvestigator software (MicroBrightField, Williston, VT, USA) on an upright Olympus BX51TRF motorized microscope (Olympus America, Center Valley, PA, USA), as described previously (18, 32). The analysis was performed blindly using five serial coronal sections spaced 15 slices apart (−1.1 to −2.9 mm A/P). The data are presented as the volume of tissue damage (mm^3^).

### Statistical analysis.

Data are presented as the standard error of the mean (SEM) and graphed using GraphPad Prism, version 10 (GraphPad Software, Inc., San Diego, CA). Comparison between two groups was performed using Student’s two-tailed t-test. Multiple comparisons between three or more groups using one-way ANOVA with post-hoc tests, and analysis across time points were performed using repeated-measures two-way ANOVA with post-hoc tests.

### Visium spatial gene expression.

Visium spatial transcriptomic analysis was performed as described by 10x Genomics (https://10xgenomics.com/). WT and KO mice were i.v. tail vein injected with 2% EBD/20% NaFl, and the animals were perfused via the heart as described previously. Brains were frozen in optimal cutting temperature (OCT) embedding medium (Fisher Healthcare, 4585) in a cryomold. The OCT-embedded tissue blocks were stored at − 80°C until cryosectioning. Mouse brains were cryosectioned at 20 μm and placed directly onto charged slides recommended by 10x Genomics. mRNA library preparation and sequencing using Illumina TruSeq by Medgenome (Foster City, CA).

### Spatial gene expression data analysis.

Reads from several capture areas (each corresponding to injured tissue, uninjured tissue, or dye regions) were aligned to the mouse reference genome (mm10-2020-A) using Space Ranger v1.3.1 (10x Genomics). The immunofluorescence images and manual alignment files were also used as input. An average of 4,981 spots under tissue per capture area (19,924 total spots) were read and processed using Partek (San Diego, CA, an Illumina company ©). High-quality spots were selected for further analysis: cells with fewer than 200 unique feature counts and hemoglobin genes with over 30% were filtered out. After spot quality control, the dataset contained 18,961 spots under tissue with 13,186 genes detected. Median ratio was used for data normalization. Linear dimensional reduction analysis was performed with PCA (1:10 PCA dimensions). Spots gene expression and clusters were visualized within UMAP projections. Regions of interest were manually selected with the interactive contour tool, and differential expression was identified using DeSeq2. Differentially expressed genes (DEGs) were identified based on Benjamini-Hochberg-corrected p-value. Gene Ontology (GO) analysis was performed using DAVID (Huang da et al., 2009a, b). We report enrichment figures focusing on biologically interpretable pathways. Therefore, the top five most significant terms were selected for figure display, except for two involving “muscle system process” and “muscle contraction”.

## Supplementary Material

This is a list of supplementary files associated with this preprint. Click to download.

• SupplementalFigures.docx

## Data Availability

The datasets analyzed during the study are available from the corresponding author upon reasonable request.

## Abbreviations

TBI: traumatic brain injury
CCI: controlled cortical impact
KO: knockout
WT: inducible wild type
i.v.: intravenous
dpi: day(s) post-injury
UMAP: uniform manifold approximation and projection
